# ATP-containing vesicles in *stria vascular* marginal cell cytoplasms in neonatal rat cochlea are lysosomes

**DOI:** 10.1038/srep20903

**Published:** 2016-02-11

**Authors:** Jun Liu, Wenjing Liu, Jun Yang

**Affiliations:** 1 Department of Otorhinolaryngology-Head and Neck Surgery, Xinhua Hospital, Shanghai Jiaotong University School of Medicine, Shanghai, China; 2 Shanghai Jiaotong University School of Medicine Ear Institute, Shanghai, China; 3 Shanghai Key Laboratory of Translational Medicine on Ear and Nose diseases, Shanghai, China; 4 Department of Otorhinolaryngology, Renji Hospital, Shanghai Jiaotong University School of Medicine, Shanghai, China

## Abstract

We confirmed that ATP is released from cochlear marginal cells in the *stria vascular* but the cell organelle in which ATP stores was not identified until now. Thus, we studied the ATP-containing cell organelles and suggest that these are lysosomes. Primary cultures of marginal cells of *Sprague-Dawley* rats aged 1–3 days was established. Vesicles within marginal cells stained with markers were identified under confocal laser scanning microscope and transmission electron microscope (TEM). Then ATP release from marginal cells was measured after glycyl-L-phenylalanine-ß- naphthylamide (GPN) treatment using a bioluminescent assay. Quinacrine-stained granules within marginal cells were labeled with LysoTracker, a lysosome tracer, and lysosomal-associated membrane protein 1(LAMP1), but not labeled with the mitochondrial tracer MitoTracker. Furthermore, LysoTracker-labelled puncta showed accumulation of Mant-ATP, an ATP analog. Treatment with 200 μM GPN quenched fluorescently labeled puncta after incubation with LysoTracker or quinacrine, but not MitoTracker. Quinacrine-labeled organelles observed by TEM were lysosomes, and an average 27.7 percent increase in ATP luminescence was observed in marginal cells extracellular fluid after GPN treatment. ATP-containing vesicles in cochlear marginal cells of the *stria vascular* from neonatal rats are likely lysosomes. ATP release from marginal cells may be via Ca^2+^-dependent lysosomal exocytosis.

ATP, an important extracellular nucleotide, is a crucial intercellular signaling molecule in both the developing^1^ and mature cochlea^2^,^3^. The diversity of the signaling pathways for this nucleotide, which includes a variety of ATP-gated channels, namely both P2X and P2Y receptor subtypes, supports a cardinal physiological role for ATP in the regulation of sound transduction, hearing sensitivity, balance, cochlear blood flow, active mechanical amplification by outer hair cells (OHC) – Deiters’ cells complex, cochlear potential, cochlear homeostasis, and vascular tension^4^,^5^,^6^.

Extracellular ATP was first reported to influence inner ear function during monitoring of the compound action potential (CAP) of the cochlear nerve and the cochlear microphonic (CM) potential as a neurotransmitter by Bobbin and Thompson in 1978^7^. Endogenous extracellular nucleosides and nucleotides were then detected in the inner ear. Muñoz’s group^8^ described low levels of ATP (10 ± 20 nM) in the endolymph and perilymph of the cochlea and reported that ATP in the perilymph increased after short-term anoxia. Furthermore, free ATP in cochlear fluids was close to that needed to cause hair cell depolarization *in vitro*. However, the source of ATP in the cochlear lymph fluids was unclear. Three possible ATP sources in the cochlea have been suggested, namely, marginal cells of the *stria vascular*^9^, supporting cells within the greater epithelial ridge of the immature cochlea^1^, and supporting cells of the mature cochlea^10^. In marginal cells, White and co-workers^11^ first reported that vesicles in these cells could be stained with quinacrine, an acridine compound that binds to nucleotides, particularly ATP. They thus suggested that these cells are possible sources of cochlear ATP, and that ATP could be secreted into the endolymph by exocytosis although evidence for this was modest. Then, Muñoz’s group^12^ provided the evidence of vesicular storage of ATP in marginal cells. Recently, we confirmed the presence of ATP and measured ATP release from the marginal cells^9^ and our work was the first to confirm that ATP release from such cells is associated with the state of the calcium pump, K^+^ channel, and activity of enzymes related to the phosphoinositide signaling pathway, such as adenylate cyclase, phospholipase C, and phospholipase A2.

However, the nature of ATP vesicles in the marginal cells was still unclear. Zhang and his colleagues^13^ reported that lysosomes in the astrocyte contain abundant ATP that can be released in a stimulus-dependent manner. Selective lysis of lysosomes abolished both ATP release and Ca^2+^ wave propagation among astrocytes, implicating physiological and pathological functions of regulated lysosome exocytosis in these cells. In addition, Wang^14^ recently reported that autophagy permits immunogenic cell death (ICD)-associated secretion of ATP, which contributes to the maintenance of lysosomal ATP stores. Furthermore, ATP release in this setting is mediated by lysosomal-associated membrane protein 1 (LAMP1) and pannexin 1 (Panx1) -dependent lysosomal exocytosis. Given that the lysosome is ubiquitous across cell types, we suppose that lysosomal vesicles and ATP vesicles depicted by White and co-workers^11^ in marginal cells of the *stria vascular* are the same, and that ATP release from the marginal cells is via Ca^2+^-dependent lysosomal exocytosis.

Next, we report that quinacrine selectively labeled lysosomes in marginal cells and confocal imaging of quinacrine- or Mant-ATP[2′-/3′-O-(N′-Methylanthraniloyl) adenosine-5′-O – triphosphate] -labeled vesicles indicated that these were lysosomes. Moreover, quinacrine-labeled electron dense precipitates within the cytoplasm in the marginal cells according to transmission electron microscopy (TEM) were identified as lysosomes. And ATP release was measured in the extracellular fluid of marginal cells after glycyl-L-phenylalanine- ß-naphthylamide (GPN) treatment. These data offered solid evidence for lysosomal ATP storage in cochlear marginal cells of neonatal rats. Our results may provide new insight into mechanisms underlying intracellular ATP storage and release in marginal cells as well.

## Results

### Primary culture of marginal cells and verification by flow cytometry

**Figure 1:**
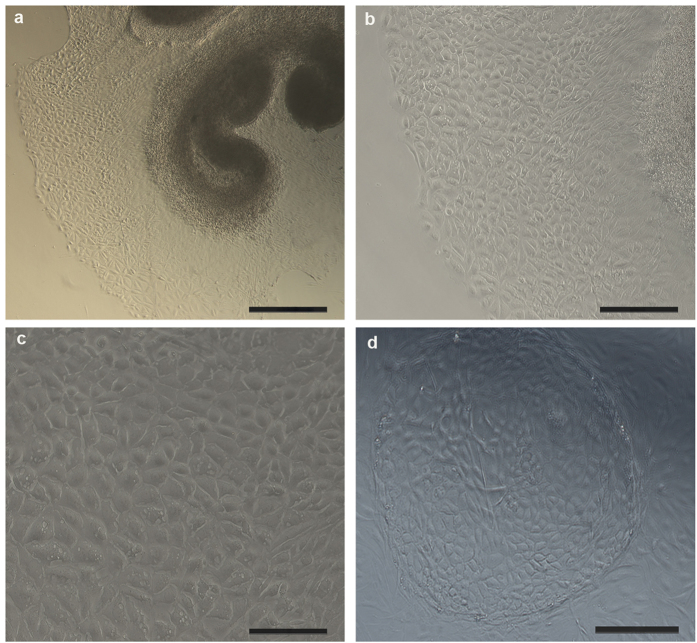
Marginal cells culture under light microscope. (**a**) Proliferated marginal cells grew outside the *stria vascular* explant and were arranged like paving stones with polygonal shape after 3 days of culture (50×), Scale bars, 400 μm. (**b**) Proliferated marginal cells grew outside the *stria vascular* explant in 3-day old cultures (100×), Scale bars, 200 μm. Larger magnification is shown in (**c**) (200×), Scale bars, 100 μm. (**d**) Proliferated marginal cells were arranged like paving stones, and formed a “cell island” in 3 day-old cultures (100×), Scale bars, 200 μm.

**Figure 2:**
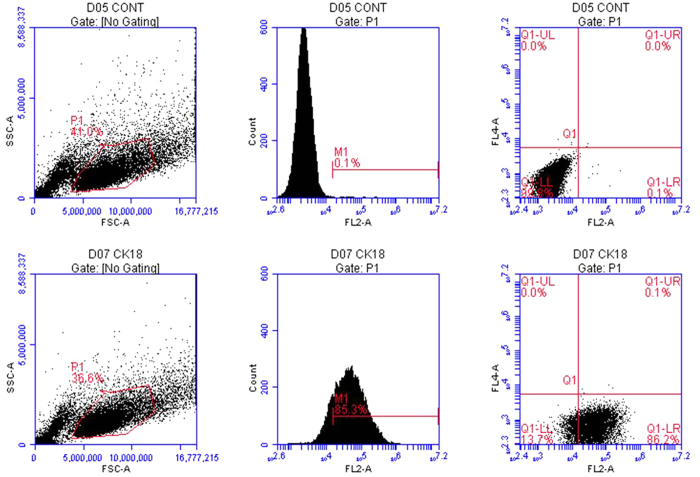
Verification of cultured marginal cells by flow cytometry. Images in the first row are marginal cells treated with FITC AffiniPure Goat Anti-Mouse IgG (H+L) (negative control). The second row contains marginal cells incubated with anti-cytokeratin 18 IgG and FITC AffiniPure Goat Anti-Mouse IgG (H+L). Flow cytometry confirmed that 85.3% of the cells were cytokeratin 18-positive cells.

We first established a primary culture of marginal cells from cochlear explants of the *stria vascular* of neonatal rats (Fig. 1). Proliferated marginal cells grew outside the *stria vascular* explant and were arranged like polygonal paving stones, with individual large nuclei. The epithelial origin of cultured marginal cells in the *stria vascular* was previously confirmed by expression of cytokeratin 18^15^. Therefore, cytokeratin 18 antibody was used to verify the purity of the cultured marginal cells in the present study. Flow cytometry revealed that 85.3% of the cells were cytokeratin18-positive cells (Fig. 2).

### Specific staining of cytoplasmic vesicles of marginal cells under confocal laser scanning microscope

**Figure 3:**
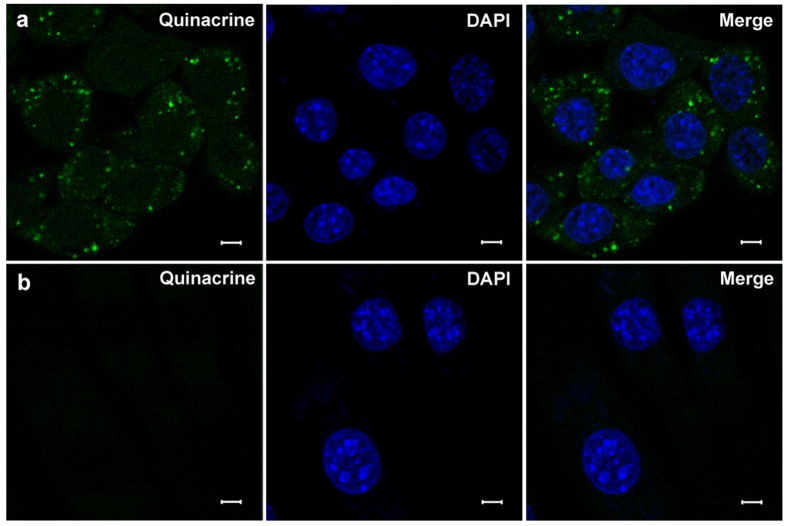
Positive staining of marginal cells and negative control 3T3 cells. Row (**a**) Left: numerous granule-like fluorescent puncta in cultured marginal cell cytoplasm incubated with quinacrine; Middle: nuclear staining with DAPI; Right: merged image of quinacrine and DAPI staining. Row (**b**) Left: The fluorescent puncta did not appear in 3T3 cells (negative control) in the cytoplasm at the same background fluorescence; Middle: nuclear staining with DAPI; Right: merged image of quinacrine and DAPI staining. Scale bars, 5 μm.

Several specific markers were used to verify vesicles within marginal cells. Incubation with quinacrine for 30 min at room temperature in the dark resulted in numerous granule-like fluorescent puncta in the cytoplasm in cultured marginal cells under confocal laser scanning microscope (Fig. 3a). Fluorescent puncta in the cytoplasm in 3T3 cells (negative control) was not observed at the same background fluorescence (Fig. 3b).

**Figure 4:**
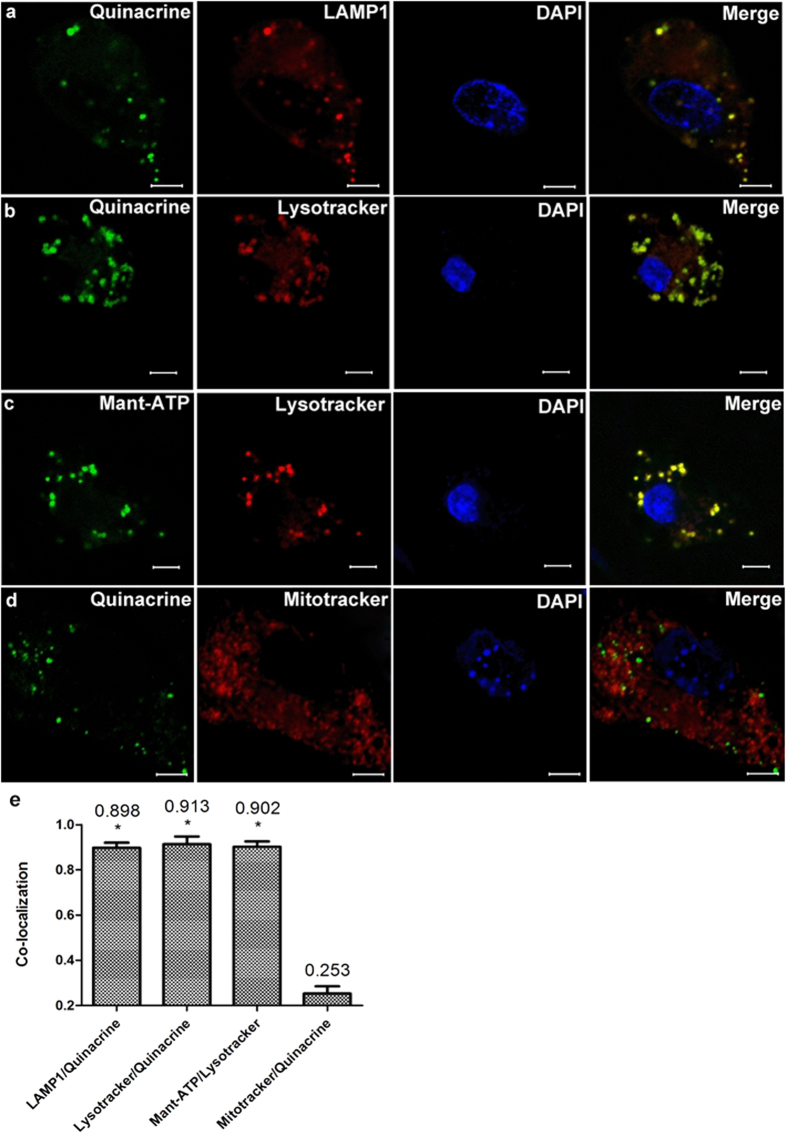
Incubation of marginal cells with specific markers for lysosomes or mitochondria. Row (**a**) Left 1: numerous green granules stained by quinacrine appeared in a marginal cell; Left 2: red granules immunostained with LAMP1 were observed in the same cell; Right 1: the cell nucleus stained with DAPI; Right 2: merged image of quinacrine, LAMP1 and DAPI cell staining indicated co-localization of quinacrine with LAMP1 puncta. Row (**b**) Left 1: green granules stained by quinacrine in a marginal cell; Left 2: red granules labeled by LysoTracker® Deep Red in the same cell; Right 1: the cell nucleus stained with DAPI; Right 2: merged image of quinacrine, LysoTracker and DAPI staining of the cell indicate co-localization of quinacrine with LysoTracker puncta. Row (**c**) Left 1: green granules stained by Mant-ATP in a marginal cell; Left 2: red granules labeled by LysoTracker® Deep Red in the same cell; Right 1: the cell nucleus stained with DAPI; Right 2: merged image of Mant-ATP, LysoTracker and DAPI staining of the cell showed co-localization of Mant-ATP with LysoTracker puncta. Row (**d**) Left 1: green granules stained by quinacrine in a marginal cell; Left 2: red granules labeled by MitoTracker® Red CMXRos in the same cell; Right 1: the cell nucleus stained with DAPI; Right 2: merged image of quinacrine, MitoTracker and DAPI staining of the cell showed no co-localization of quinacrine with MitoTracker puncta. Scale bars, 5 μm. (**e**) Summary of the co-localization of quinacrine or Mant-ATP with different specific markers. The number above each column refers to mean co-localization coefficient. 5 images obtained from 5 independent experiments were calculated for co-localization analysis in each group, error bars indicate SD. (*P < 0.01 compared with Mitotracker/Quinacrine, independent samples *t*-test). The merge images of quinacrine/LAMP1, quinacrine/LysoTracker or Mant-ATP /LysoTracker showed a high proportion of co-localization.

Then, marginal cells loaded with quinacrine (green) were immunostained with LAMP1 (red) (Fig. 4a), a specific marker for lysosomes^16^. Average 89.8% of quinacrine-stained granules were confirmed to be immunopositive for LAMP1, indicating the co-localization of quinacrine- and LAMP1-positive puncta (n = 5, Fig. 4e).

When marginal cells were incubated with quinacrine (green) and labeled with LysoTracker® Deep Red (red), the lysosome tracer, the co-localization of green and red was observed (Fig. 4b,e). LysoTracker-labelled puncta also showed accumulation of fluorescent ATP analog, Mant-ATP (green) (Fig. 4c,e). While marginal cells labeled with MitoTracker® Red CMXRos (red), the mitochondria tracer, had not shown the co-localization of green and red after incubation with quinacrine (green) (Fig. 4d,e).

### Selectively disrupting lysosomes or mitochondria in cultured marginal cells

**Figure 5:**
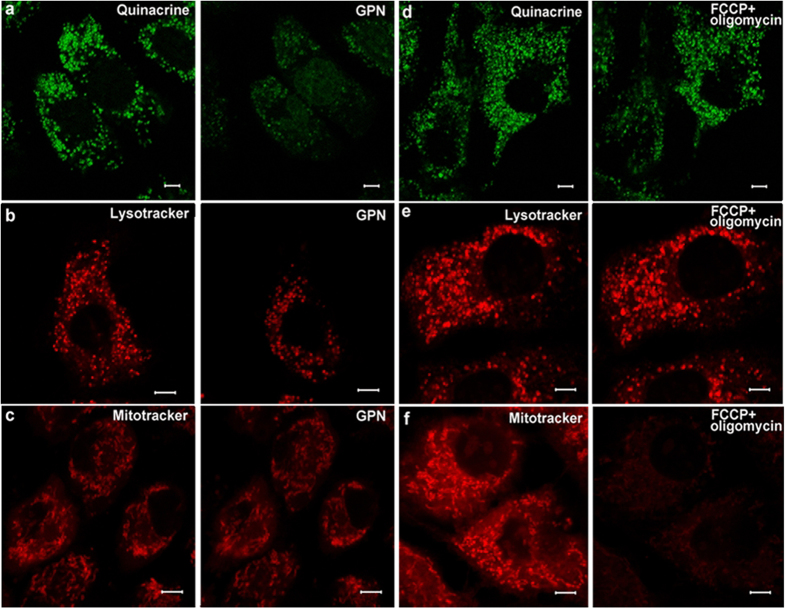
Images of puncta labeled by dyes treated with 200 μM GPN or FCCP (1 μM) + oligomycin (10 μM) for 15 min. Row (**a**) Left: green fluorescent punctas in the cytoplasm in a cultured marginal cell incubated with quinacrine; Right: Quinacrine stained puncta was quenched after treatment with 200 μM GPN for 15 min. Row (**b**) Left: red punctas in a cultured marginal cell after incubation with LysoTracker® Deep Red; Right: LysoTracker stained puncta within the cell was attenuated after treatment with 200 μM GPN for 15 min. Row (**c**) Left: red punctas revealed mitochondria in cultured marginal cells incubated with MitoTracker® Red CMXRos; Right: Red fluorescence did not change after treatment with 200 μM GPN for 15 min. Row (**d**) Left: green fluorescent punctas in the cytoplasm in cultured marginal cells incubated with quinacrine; Right: Quinacrine stained puncta did not change in cells after treatment with FCCP(1 μM) + oligomycin (10 μM) for 15 min. Row (**e**) Left: red punctas in cultured marginal cells after incubation with LysoTracker® Deep Red; Right: LysoTracker stained puncta within the cell did not change after treatment with FCCP(1 μM) + oligomycin (10 μM). Row (**f**) Left: red punctas revealed mitochondria in cultured marginal cells incubated with MitoTracker® Red CMXRos; Right: red punctas vanished after treatment with FCCP(1 μM) + oligomycin (10 μM).

After incubation with quinacrine or LysoTracker® Deep Red, treatment with 200 μM GPN, a substrate of the lysosomal exopeptidase cathepsin C that selectively induces lysosome osmodialysis^17^,^18^,^19^, largely attenuated the appearance of labeled puncta within the marginal cells, however, this did not occur when staining with MitoTracker® Red CMXRos (Fig. 5a–c).

In contrast, treatment with *p*-trifluoromethoxyphenylhydrazone (FCCP, 1 μM) and oligomycin (10 μM), specific toxins that disrupt mitochondria, weakened fluorescent labeling from MitoTracker® Red CMXRos, but did not influence staining of ATP vesicles by quinacrine or lysosome staining by LysoTracker® Deep Red (Fig. 5d–f).

### Lysosomal exocytosis in marginal cells

**Figure 6:**
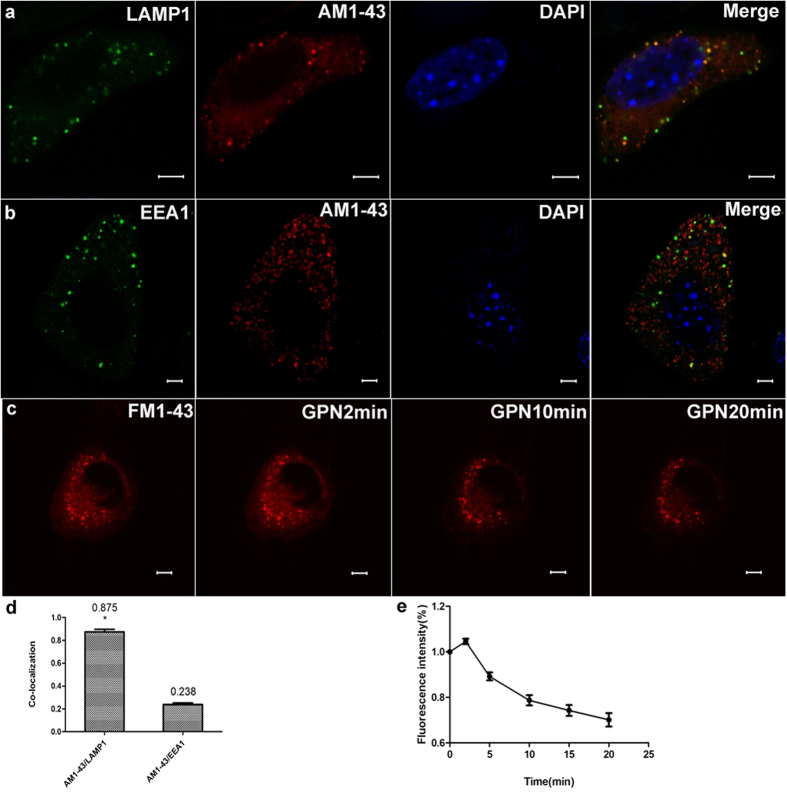
Incubation of marginal cells with FM dyes selectively labels lysosomes and GPN-evoked lysosomal exocytosis in marginal cells. Row (**a**) Left 1: green granules immunostained with LAMP1 appeared in a marginal cell; Left 2: red granules labeled by AM1-43 were observed in the same cell; Right 1: the cell nucleus stained with DAPI; Right 2: merged image of LAMP1, AM1-43 and DAPI cell staining indicated co-localization of LAMP1 and AM1-43 puncta. Row (**b**) Left 1: green granules immunostained with EEA1 appeared in a marginal cell; Left 2: red granules labeled by AM1-43 were observed in the same cell; Right 1: the cell nucleus stained with DAPI; Right 2: merged image of LAMP1, AM1-43 and DAPI cell staining indicated no co-localization of EEA1 and AM1-43 puncta. Scale bars, 5 μm. Row (**c**) Left1: red fluorescent punctas in the cytoplasm in a cultured marginal cell incubated with FM1-43; Left 2: increased staining was monitored in 2 minutes after GPN stimulation; Right 1: decreased staining was monitored after treatment with 200 μM GPN for 10 min. Right 2: decreased staining was monitored after treatment with 200 μM GPN for 20 min. Scale bars, 5 μm. (**d**) Summary of the co-localization of AM1-43 with different specific markers. The number above each column refers to mean co-localization coefficient. 5 images obtained from 5 independent experiments were calculated for co-localization analysis in each group, error bars indicate SD. (*P < 0.01 compared with AM1-43/EEA1, independent samples *t*-test). The merge images of AM1-43/LAMP1 showed a high proportion of co-localization. (**e**) Summary of fluorescence intensity of marginal cells incubated with FM1-43 after GPN stimulation. n = 5, error bars indicate SD.

AM1-43, a fixable FM1-43 analogue, labeled average 87.5% population of vesicles that were immunopositive for the lysosomal membrane protein LAMP1 (n = 5, Fig. 6a,d), but not for the early endosome marker EEA1^13^,^20^ (Fig. 6b,d). To further characterize the lysosomal exocytosis in marginal cells, we incubated cells with fluorescent FM1-43 dyes, which selectively label the vesicles exhibiting functional exocytosis through the endocytosis-exocytosis recycling pathway^21^,^22^, increased staining was monitored after 2 minutes of GPN stimulation, then decreased staining was monitored (n = 5, Fig. 6c,e).

### TEM

**Figure 7:**
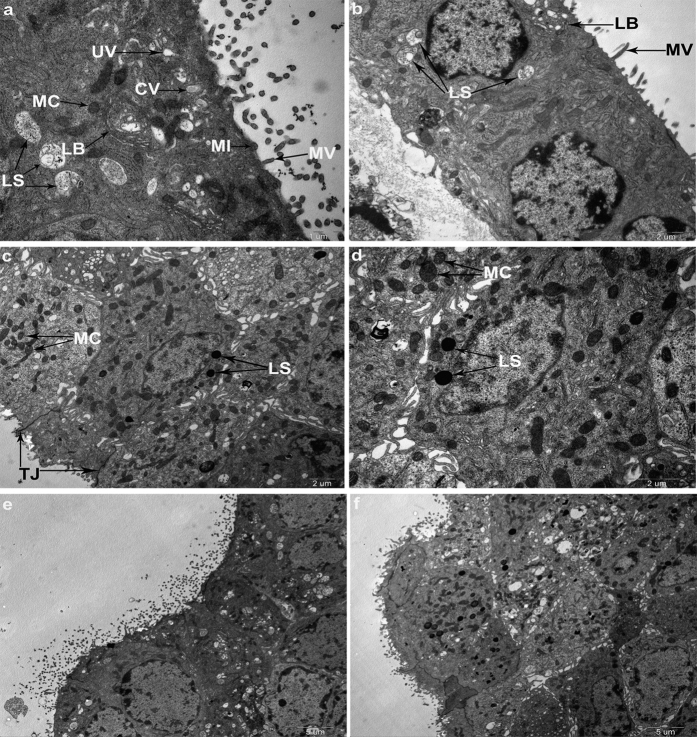
TEM revealed characteristics of marginal cells and lysosomal exocytosis. (**a**) TEM photograph shows characteristics of the marginal cell. Arrows indicate lysosomes (LS), mitochondria (MC), coated vesicles (CV), uncoated vesicles (UV), small invaginations (MI), microvilli-like extensions (MV), lamellar bodies (LB), respectively. Scale bars, 1 μm. (**b**) Marginal cells under TEM. Arrows indicate LS, MV and LB, respectively. Scale bars, 2 μm. (**c,d**) TEMs of marginal cells loaded with quinacrine for 30 min. Arrows indicate quinacrine labeled lysosomes and unlabeled mitochondria. Neighboring cells were connected with tight junctions. Scale bars, 2 μm. (**e,f**) Lysosomal exocytosis was observed in marginal cells that contained granules stained with quinacrine. TEM showed the characteristics of the marginal cells and lysosomal exocytosis. A coated omega-shaped invagination was found in the apical plasma membrane of the marginal cells (**e**). Scale bars, 5 μm.

The ultra-structure of the cultured marginal cells was observed by TEM which revealed many microvilli-like extensions, numerous coated and uncoated vesicles, coated omega-shaped invaginations, quinacrine labeled lysosomes, and unlabeled mitochondria (Fig. 7). Neighboring cells were connected with tight junctions. Several coated omega-shaped invaginations that may represent regions of active transport through exo- or endocytosis were found in the apical plasma membrane.

### ATP release from cultured marginal cells after GPN treatment or Triton X-100 and Ca^2+^ dependence

**Figure 8:**
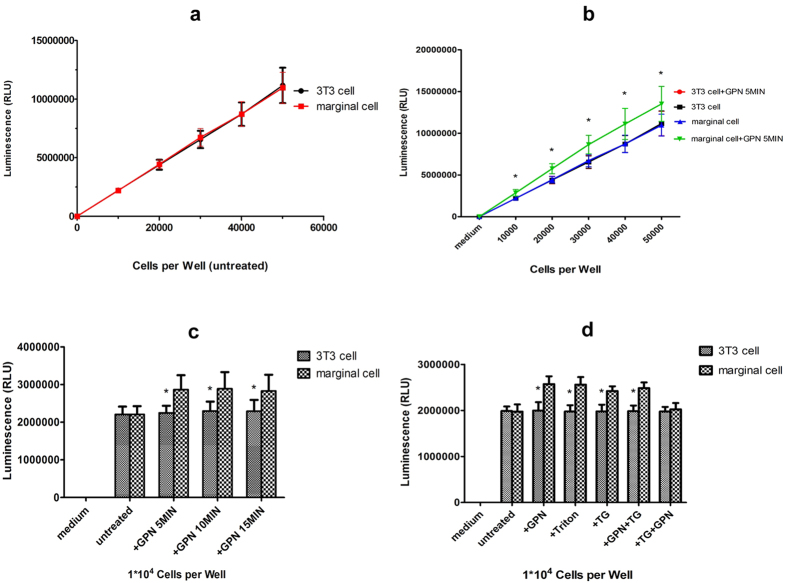
ATP release from marginal cells after treatment with GPN, Triton, TG, GPN + TG. (**a**) A direct relationship between luminescence kit and the number of 3T3 cells (r1) or marginal cells (r2) from 0 to 50,000. r1^2^ = 0.9996, r2^2^ = 0.9997 (**b**) Treatment with 200 μM GPN resulted in an increase of average 27.7% of luminescence in serial fivefold dilutions of marginal cells compared with 3T3 control (n = 12, *P < 0.01, independent samples *t*-test). Error bars indicate SD. (**c**) Time course of ATP release in marginal cells and 3T3 cells after 200 μM GPN exposure (n = 12, *P < 0.01, independent samples *t*-test). Error bars indicate SD. (**d**) Respective treatment with different reagents (200 μM GPN, 1% Triton X-100, 10 μM TG, 200 μM GPN + 5 μM TG) for 5 min resulted in ATP release from marginal cells compared with 3T3 control (n = 12, *P < 0.01, independent samples *t*-test). However, GPN (200 μM) did not cause ATP release from marginal cells compared with 3T3 control after TG treatment (5 μM) for 5 min (n = 12, *P* > 0.05, independent samples *t*-test). Error bars indicate SD.

To detect ATP release from cultured marginal cells, a direct linear relationship (r1^2^ = 0.9996 for 3T3 cells; r2^2^ = 0.9997 for marginal cells) was confirmed between luminescence measured with the assay kit and the fivefold dilutions of cells varied from 0 to 50,000 (Fig. 8a). 200 μM GPN treatment resulted in an average increase of 25.7% in luminescence in serial fivefold dilutions of marginal cells compared with 3T3 control (n = 12, *P < 0.01, independent samples *t*-test) (Fig. 8b). Together with given that GPN can disrupt lysosomes in marginal cells, suggesting that these lysosomes most likely contained ATP.

ATP release in marginal cells was greater than 3T3 controls after 200 μM GPN treatment for 5, 10, and 15 min respectively (n = 12, *P < 0.01, independent samples *t*-test). Interestingly, ATP release in marginal cells was not different at any time point after GPN treatment (n = 12, *P > 0.05, independent samples *t*-test) (Fig. 8c). In addition, the high concentration of ATP was detectable after 5 min of treatment with 1%Triton X-100. Application of 10 μM thapsigargin (TG), the endoplasmic reticulum (ER) calcium store inhibitor, also caused ATP release. However, GPN did not increase ATP release after 5 μM TG treatment for 5 min (n = 12, *P < 0.01, independent samples *t*-test (Fig. 8d).

## Discussion

First, we established a primary culture of marginal cells from cochlear explants of the *stria vascular* area in neonatal rats. The *stria vascular* was isolated by microdissection, and dissociated into single cells after tissue incubation for three days. Then marginal cells were purified by trypsin digestion combined with differential adhesion methods. As early as 1989, Rarey and Patterson^23^ established primary cell culture of bovine *stria vascular* using dissociated cell techniques. In this study, ten days were required to purify marginal cells in our group compared to 14 days required by Kim’s group^15^. Separation of the *stria vascular* from the spiral ligament is difficult and marginal cells proliferated toward the apical surface of the explant quickly in the first 3 days of tissue culture. Therefore, we developed a tissue culture technique that can simulate an *in vivo* environment and allows marginal cell growth. Specifically, because fibroblasts grew rapidly on the 4^th^ day of culture, 0.25% trypsin was used to digest fibroblasts and then a sterile 0.1% collagenase (type I) solution was used to dissociate the culture into single cells. The differential adhesion method widely used in primary cell cultures^24^,^25^,^26^ was modified at the purification step which was repeated several times until purified marginal cells were obtained.

Vesicular storage of ATP in cochlear *stria vascular* marginal cells has been reported and ATP release through exocytosis in human monocytes^27^, astrocytes^13^ and microglial cells^20^ has been documented. Moreover, ATP release from marginal cells was detected in the previous report^9^. Therefore, in the present study, we explored lysosomal ATP stores in marginal cells of the *stria vascular* in the cochlea of neonatal rats. Staining of isolated marginal cells with quinacrine, a putative marker of ATP and an acridine derivative which reversibly binds to adenine nucleotide and DNA at a ratio of 1 quinacrine molecule to 4 nucleotides^28^,^29^,^30^, revealed numerous green granule-like fluorescent puncta in the cytoplasm under confocal laser scanning microscopy. Furthermore, much of the co-localization was observed with double staining of quinacrine/LAMP1, quinacrine/LysoTracker or Mant-ATP/LysoTracker in which LAMP1 and LysoTracker are specific markers for lysosomes, while Mant-ATP is a fluorescent nucleotide analogue used for studying ATP stores and nucleotide-binding proteins^13^,^20^. Quinacrine-stained vesicles did not pick up the tracer dye of the mitochondria which is one of the potential sources of ATP in the cells. Moreover, TEM revealed that only lysosomes were labeled by quinacrine. A high concentration of ATP was detectable after treatment for 5 min with 1% Triton X-100, indicating that ATP was contained within membrane-enclosed compartments. Furthermore, treatment of marginal cells for 5 min with 200 μM GPN, a reagent that selectively induces lysosome osmodialysis, increased ATP in the extracellular fluid in a Ca^+^-dependent manner. Collectively, these results indicate that the vesicles containing ATP are likely lysosomes.

Ultra-structural characteristics of marginal cells under TEM have been reported^15^,^31^,^32^,^33^. Numerous cytoplasmic coated (CV) and uncoated vesicles (UV), lamellar bodies (LB) of unknown origin have been observed in our work. We suggested these objects may be different stages of lysosomes during cell metabolism. ATP has been reported to be secreted by exocytosis in nerves, human monocytes, astrocytes and microglial cells^13^,^20^,^27^,^34^. Coated omega-shaped invaginations representing regions of active transport were found in the apical plasma membrane, indicating exo- or endocytosis. The presence of lysosomal stores and the apparent abundant vesicular structures observed at the level of the luminal surface of the marginal cells of the *stria vascular* might imply that ATP was actively transported across the membrane of these cells. Therefore, it is likely that ATP release from marginal cells is through exocytosis.

To further characterize the lysosomal exocytosis in marginal cells, fluorescent FM1-43 dye was incubated with isolated cells. The FM1-43 dyes contain a cationic head, making them impermeable to membranes. When a secretory cell is stimulated to evoke exocytosis, FM1-43 molecules that were inserted in the membrane are internalized during compensatory endocytosis and newly formed secretory granules or vesicles become stained with dye (staining/endocytosis). If stained secretory granules or vesicles undergo exocytosis in dye-free medium, due to concentration gradient, FM1-43 molecules dissociate from the membrane and loose fluorescence (destaining/excocytosis)^21^,^22^,^35^. In our work, increased fluorescence was observed after 2 minutes of GPN stimulation, following with attenuated staining. This recycling of secretory membrane could be monitored as an indication of exocytosis after treatment of GPN^21^,^22^. Furthermore, we found that AM1-43, a fixable FM1-43 analogue that is largely preserved after immunochemical procedures, labeled about 87.5% population of vesicles that were immunopositive for the lysosomal membrane protein LAMP1, but not for the endosomal marker EEA1. Thus, we suggest that an increased extracellular ATP resulting from treatment of cells with GPN is caused by lysosomal exocytosis. Although the mechanism of GPN-induced increase in extracellular ATP remains unknown, it should not be ruled out that ATP leak from cytoplasm is due to cell damage in this case. More experiments to confirm the ATP release pathways are needed in the future.

Reportedly, Ca^2+^ signaling gives rise to disruption of lysosomes by exposure to GPN in human monocytes^27^. Zhang’s group demonstrated that calcium wave propagation is the most regulator of lysosomal exocytosis in astrocytes that contain abundant ATP^13^. In our previous work^9^, one of the important characteristics of ATP release from marginal cells was found to be Ca^2+^ dependent. In the present study, application of the ER calcium store inhibitor, TG, increased extracellular ATP. However, GPN was unable to increase extracellular ATP following TG treatment. This result suggests an interaction exists between ER and lysosomal compartments in marginal cells and ATP release from these cells is Ca^2+^ dependent as well. TG is an inhibitor of the Ca^2+^-ATP enzyme, responsible for replenishing Ca^2+^ into the ER. Sivaramakrishnan *et al.*^27^ demonstrated that bidirectional communication exists between lysosomal and ER Ca^2+^ stores via three pathways: (i) release of ER Ca^2+^ triggers lysosome exocytosis (ER to lysosome); (ii) activation of ryanodine receptors (lysosome to ER) and (iii) release of ATP and mobilisation of ER calcium through activation of PLC-coupled P2Y receptors (lysosome to ER). Ying Dou *et al.*^20^ reported that microglia cells release ATP through Ca^2+^ dependent lysosomal exocytosis. Our work may suggest that GPN induces Ca^2+^ leak from lysosomes and an increased intracellular Ca^2+^ subsequently causes lysosomal exocytosis, thus intracellular ATP was released to the outside of the cell. The extracellular ATP activates cell surface P2Y receptors to generate Ins(1,4,5)P3(IP3) and bind to IP3 receptors, causing ER Ca^2+^ release. Since present results are consistent with those previously reported, we speculated that ATP release from marginal cells may be via Ca^2+^-dependent lysosomal exocytosis.

The mechanism underlying ATP accumulation in the lysosomes is unknown. An acidic luminal pH (pHL) in lysosomes must be maintained to activate hydrolytic enzymes and to degrade internalized macromolecules^36^,^37^. Protons are pumped into the lumen of the lysosomes, which uses energy from ATP hydrolysis through a vacuolar-type H^+^-ATPase. ATP is negatively charged and ATP-binding-cassette (ABC) proteins are expressed on the lysosome membrane^38^. High-level activity of the proton pump in the lysosome membrane may maintain an even higher positive membrane potential in the lysosome, allowing lysosomes to accumulate more ATP through ABC transporters.

In conclusion, ATP-containing vesicles in cochlear marginal cells of the *stria vascular* from neonatal rats are likely lysosomes. ATP release from marginal cells may be via Ca^2+^-dependent lysosomal exocytosis.

The limitation of this study is that we could not identify ATP-containing vesicles from secretory vesicles, one kind of membranous vesicular organelles that perform a variety of biological functions ranging from secretion to cellular communication in eukaryotic cells^39^ though all the evidence here points to ATP-containing vesicles as lysosomes. More experiments to address this issue are needed in the future.

## Methods

### Marginal cells culture

Male and female neonatal *Sprague-Dawley* rats (1–3 days-of-age) were provided by the Shanghai Laboratory Animal Center of the Chinese Academy of Sciences. All parents of neonatal rats were tested for positive auricle reflexes. Rats were euthanized using a rapid guillotine method, approved by the Animal Care Committee. Cochlea was removed from the surrounding bone, and the outer coil containing the *stria vascular* and spiral ligament was isolated by microdissection in Dulbecco’s phosphate-buffered saline (DPBS, pH 7.35, GIBCO). Isolated tissues were then plated on poly-L-lysine coated dishes with the marginal cell surface facing up. Cultures were maintained with growth medium: RPMI-1640 culture medium (Hyclone, NYL1024) was supplemented with 10% fetal bovine serum (FBS, GIBCO, 10099-141, Australia), and 100 U/ml penicillin (GIBCO) in an incubator (Thermo Scientific HERA CELL 150i CO_2_ incubator) with 5% CO_2_ at 37^o^C.

After three days of culture, 0.25% trypsin (1×) (GIBCO, 15050-065) was used to digest fibroblast cells, and then a sterile 0.1% collagenase (type I) (Sigma, C-0130) solution (w/vol; in serum-free 1640 culture medium) was used to dissociate the culture into single cells for 15 min in an incubator with 5% CO_2_ at 37 °C. Cells were harvested by centrifugation at 1,000 × g for 3 min, then re-suspended in 10 ml growth medium and cultured in a 100 mm^3^ culture plate in an incubator. Dead and non-adherent cells were removed by refreshing the culture medium after 12 h of culture. Culture medium was refreshed every 2 days and purified marginal cells were obtained after seven days of cell culture by 0.25% trypsin (1×) (GIBCO, 15050-65) and 0.25% trypsin-EDTA (1×) (GIBCO, 25200-056) digestion combined with differential adhesion methods. Adherent cells were tentatively identified as marginal cells of the cochlear *stria vascular*.

All animal experiments were approved by Shanghai Jiaotong University School of Medicine, and the experimental methods were carried out in accordance with the approved guidelines by Institutional Animal Care and Use Committee of Shanghai Jiaotong University School of Medicine (License No. (Shanghai): 2008-0052).

### Verification of cultured marginal cells by flow cytometry

Marginal cells were harvested and re-suspended (1 × 10^6^ cells/ml) in ice cold PBS and dissociated cells were incubated with anti-cytokeratin 18 IgG (1:200 dilution, Abcam, ab82254) for 1 h at room temperature in the dark after 3% FBS treated for 30 min. Then, cells were incubated for 30 min at room temperature in the dark with secondary antibodies in 3% FBS (1:200 dilution, FITC AffiniPure Goat Anti-Mouse IgG (H+L), EarthOx, San Francisco, California, E031210-01). Cells were washed three times by centrifugation at 1,000 × g for 3 min after antibody incubation and re-suspended in 0.5 ml 1 × PBS. The relative proportion of cells with fluorescent surfaces was measured with flow cytometry (Accuri™ C6, Becton, Dickinson and Company, NewYork, State of NewYork).

### Confocal laser scanning microscopy

Marginal cell suspension aliquots were stained with quinacrine dihydrochloride (5 × 10^−6^ mol/L, Sigma Chemicals, Perth, Western Australia, 1 × PBS) for 30 min at room temperature in the dark and observed under a confocal laser scanning microscopy (ZEISS LSM710, Germany). 3T3 cells, a fibroblast cell line, were purchased from America Type Culture Collection (ATCC, Manassas, VA) and were used as a negative control in this study. Immunohistochemical and double immunofluorescence techniques included loading marginal cells with quinacrine dihydrochloride (5 × 10^−6^ mol/L) and immunostaining with anti-LAMP1 antibody (1:200 dilution, Abcam, ab24170), Dylight 594, Goat Anti-Rabbit IgG (H+L) secondary antibody (1:500 dilution, EarthOx, E032420-01) and DAPI (Sigma, D-9542) was used for nuclear staining (50 μg/ml). Marginal cells were incubated with quinacrine (5 × 10^−6^ mol/L) for 30 min and then incubated with Lyso-tracker (100 nmol/L, Invitrogen, L12492) or Mito-tracker (100 nmol/L, Cell Signaling Technology, M9082) for 60 min at room temperature in the dark, and the nucleus was stained with DAPI for 10 min. Marginal cells were cultured with fluorescent ATP analog, Mant-ATP(300 μM, Anaspec, AS-64610) for 5 h after being treated with Lyso-tracker for 60 min. Marginal cells were loaded with quinacrine for 30 min, Lyso-tracker, or Mito-tracker was used for 60 min at room temperature in the dark, and then cells were treated with glycyl-L-phenylalanine-ß-naphthylamide (GPN, 200 μM, Cayman Chemical,14634) or *p*-trifluoromethoxyphenylhydrazone (FCCP, 1 μM, Sigma, C2920) and oligomycin (10 μM, Merck Millipore, CAS 1404-19-9). Marginal cells were loaded with 2.5 μM fluorescent FM1-43(Biotium, 70020) for 10 min at 37 ^o^C and then cells were treated with GPN (200 μM). Marginal cells were incubated with 10 μM AM1-43(Biotium,70024) and immunostained with anti-LAMP1 antibody (1:200 dilution, Abcam, ab24170) or anti-EEA1 antibody(1:200 dilution, Abcam, ab2900), then loaded with Dylight 488, Goat Anti-Rabbit IgG (H+L) secondary antibody (1:500 dilution, EarthOx, E032220) and DAPI was used for nuclear staining. Marginal cells were washed 3 times in PBS before being transferring to chambers for imaging under a confocal microscope with a 63 × oil-immersion objective. Fluorescent images of quinacrine (green) and Mant-ATP (green) or DAPI (blue) were obtained with λ_excitation_ = 488 nm, λ_excitation_ = 720 nm or λ_excitation_ = 405 nm, respectively. Fluorescent images of Lyso-tracker (red), Mito-tracker (red) and LAMP1 (red) were obtained with λ_excitation_ = 594 nm. Fluorescence of FM1-43(red) and AM1-43 (red) were detected at 560–625nm with excitation at 488 nm. Fluorescent images of LAMP1 (green) and EEA1 (green) were obtained with λ_excitation_ = 488 nm. A low laser power (less than 0.5% power) was used to avoid possible fluorescent bleaching. A decrease in fluorescent intensities of quinacrine due to photobleaching was less than 5% over 10 min. A total of 5 images obtained from 5 independent experiments were calculated for co-localization analysis in each group. Data analysis was performed with Image Pro Plus 6.0 (Media Cybernetics), SPSS 20.0 software (IBM SPSS Inc., NewYork, State of NewYork) and GraphPad Prism v5.0 (GraphPad Software, Inc., Santiago, California); error bars indicate SD.

### TEM

Marginal cell suspension aliquots were stained with quinacrine dihydrochloride (5 × 10^−6^ mol/L; 1 × PBS) for 30 min at room temperature in the dark. Then, fractions were fixed by the slow addition of 4% glutaraldehyde in 0.1 M PBS for more than 2 h at room temperature in the dark. Marginal cells were post-fixed in 1% O_s_O_4_ and dehydrated in ascending concentrations of ethanol and embedded in epoxy resin within the microcentrifuge tube. Sections were then cut with a diamond knife, stained with a saturated solution of uranyl acetate in 50% ethanol and lead citrate. Sections were examined and photographed with a Philips CM120 electron microscope at 80 KV.

### ATP measurement

To measure ATP release from marginal cells, an ATP bioluminescence assay kit (Promega, G7570) was used along with an opaque 96-well plate (Coring, 3912) to avoid optical cross-talk. Control wells containing culture medium without cells were used for background luminescence and 100 μl samples per well were withdrawn at room temperature. Clarified samples were mixed 1:1 with luciferase reagent before measurements were made using a Modulus Luminometer (Centro XS^3^ LB 960, BERTHOLD) with 1 s integration time. The contents were mixed for 2 min on an orbital shaker to induce cell lysis. Luminescence of 3 parallel replicates was recorded of each cell group and every experiment was repeated four times. Luminescent values represent means ± SD of 12 replicates for each cell group. After 200 μM GPN was added to the 5 different dilutions of marginal cells, luminescence was measured before or 5, 10, or 15 min after stimuli, respectively. Treatment with 200 μM GPN, 1% Triton X-100 (Sigma, T9284), 10 μM TG (Sigma, T9033), 200 μM GPN + 5 μM TG and 5 μM TG + 200 μM GPN was performed in another experiment with 10,000 cells per well and 3T3 cells were controls. Data represent averaged results from at least four experiments for each group. Data analysis was performed with SPSS 20.0 software (IBM SPSS Inc., NewYork, State of NewYork) and GraphPad Prism v5.0 (GraphPad Software, Inc., Santiago, California); error bars indicate SD.

## Conclusions

Vesicles containing ATP are likely lysosomes in marginal cells of the *stria vascular* in neonatal rat cochlea. ATP within these vesicles may be transported across membranes through Ca^2+^-dependent lysosomal exocytosis.

## Additional Information

**How to cite this article**: Liu, J. *et al.* ATP-containing vesicles in *stria vascular* marginal cell cytoplasms in neonatal rat cochlea are lysosomes. *Sci. Rep.* **6**, 20903; doi: 10.1038/srep20903 (2016).
